# Low-Density Granulocyte Contamination From Peripheral Blood Mononuclear Cells of Patients With Sepsis and How to Remove It – A Technical Report

**DOI:** 10.3389/fimmu.2021.684119

**Published:** 2021-08-18

**Authors:** Judith Schenz, Manuel Obermaier, Sandra Uhle, Markus Alexander Weigand, Florian Uhle

**Affiliations:** Department of Anesthesiology, Heidelberg University Hospital, Heidelberg, Germany

**Keywords:** cell isolation, centrifugation, Ficoll, MACS, density gradient, Sepsis, PBMC (peripheral blood mononuclear cells), low-density granulocytes

## Abstract

Elucidating the mechanisms contributing to the dysregulated host response to infection as part of the syndrome is a current challenge in sepsis research. Peripheral blood mononuclear cells are widely used in immunological studies. Density gradient centrifugation, a common method, is of limited use for blood drawn from patients with sepsis. A significant number of low-density granulocytes co-purify contributing to low purity of isolated peripheral blood mononuclear cells. Whole blood anticoagulated with lithium heparin was drawn from patients with sepsis (n=14) and healthy volunteers (n=11). Immediately after drawing, the plasma fraction was removed and PBMC were isolated from the cellular fraction by density gradient centrifugation. Samples derived from patients with sepsis were subsequently incubated with cluster of differentiation 15 MicroBeads and granulocytes were depleted using magnetic-activated cell sorting. Core cellular functions as antigen presentation and cytokine secretion were analyzed in cells isolated from healthy volunteers (n=3) before and after depletion to confirm consistent functionality. We report here that depleting CD15^+^ cells after density gradient centrifugation is a feasible way to get rid of the low-density granulocyte contamination. Afterwards, the purity of isolated, functionally intact peripheral blood mononuclear cells is comparable to healthy volunteers. Information on the isolation purity and identification of the containing cell types are necessary for good comparability between different studies. Depletion of CD15^+^ cells after density gradient centrifugation is an easy but highly efficient way to gain a higher quality and more reliability in studies using peripheral blood mononuclear cells from septic patients without affecting the functionality of the cells.

## Introduction

The currently valid sepsis definition, published in 2016, focuses on the dysregulated host response to infection leading to a life-threatening organ dysfunction (1). Despite great efforts in recent years, many of the mechanisms contributing to this dysregulation are still not properly understood. A deeper insight into the underlying immunopathology is of utmost importance to develop new therapy strategies and consequently a targeted patient care (2). In addition, it is also necessary to determine sepsis sub-groups (endotypes) according to their clinical and outcome characteristics within the very heterogeneous syndrome (3). Currently, different sepsis endotypes are identified through clinical variables or blood gene expression profiles (4–6). Expanding this concept, a further characterization of these already identified endotypes based on immunological parameters as well as the identification of new endotypes based on such parameters is possible. This ensures that misperceptions made in the past based on trials conducted on unstratified patient cohorts are not repeated (7), but patients benefit from precision medicine.

Within this framework, a substantial amount of research is conducted using peripheral blood mononuclear cells (PBMC), defined as lymphocytes and monocytes, from septic patients’ blood due to the ease of isolation by density gradient centrifugation. However, even the best designed studies lose their biological validity if based on wrong methodological assumptions. In this article we would therefore like to draw attention to a phenomenon that has been known for a long time but has received little attention: In certain pathological conditions apart from sepsis (8) including burn patients (9, 10), chronical infections (11, 12), and autoimmune diseases (13), but also in healthy pregnancies (14), increased amounts of granulocytes with a lower density compared to normal neutrophils can be found. The detailed elucidation of their origin, function, and role in these conditions is part of current research efforts (15). As their density resembles those of lymphocytes and monocytes, classical density gradient centrifugation with the aim of isolating PBMC results in a relevant low-density granulocyte contamination, which is expected to strongly bias down-stream analytical methods. Here, we provide a simple, yet highly effective solution to improve preanalytical isolation of PBMC from patients with sepsis.

## Materials and Methods

### Study Cohort

The study protocol of the clinical study was assessed and positively evaluated by the ethics committee of the Medical Faculty of the Heidelberg University, Germany (S-003/2018). The study was registered in the German Clinical Trials Register (ID: DRKS00018867) and conducted in the interdisciplinary surgical intensive care unit of the Heidelberg University Hospital. This publication reports on a subcohort of the full study. All patients with sepsis and healthy volunteers were of legal age and gave written informed consent. If the patients were incapable, it was obtained from their legal designees. For enrolment of patients, at least two SIRS criteria (16) had to be fulfilled, clinical or microbiological proof of infection as well as sepsis-associated organ dysfunction (1) (defined as a change in the Sequential Organ Failure Assessment (SOFA) score by at least two points compared to the previous day) were needed (**Table 1**). Exclusion criteria were: sepsis diagnosis >24h, pregnancy, enrolment in an interventional study, immunosuppression, or viral infections. Blood was drawn at enrolment [sepsis onset (d1)] and, if possible, at day 8 (d8).

**Table 1:** Study cohort.

	Patients with sepsis (n=14)	Healthy subjects (n=11)
Age, years	63 (38 - 82)	41 (25 - 58)
Sex, male/female	10 (71%)/4 (29%)	8 (73%)/3 (27%)
SOFA score at admission	10 (7 - 15)	
Infection focus (multiple selection possible)		
Abdominal	12 (86%)	
Pulmonary Urogenital	5 (36%) 2 (14%)	

Data are presented as median (range) for age and SOFA score or as absolute number (percentage) in case of gender and infection focus.

### PBMC Isolation

PBMC were isolated by density gradient centrifugation. In detail, 30mL whole blood anticoagulated with lithium heparin was drawn and processed immediately. After centrifugation (2,000x*g*, 10min), the plasma fraction was removed. The cellular fraction was resuspended in 30mL phosphate-buffered saline (PBS) (Thermo Fisher Scientific, Waltham, USA), transferred into two Leucosep™ tubes pre−filled with separation medium for PBMC isolation (greiner bio-one, Kremsmuenster, Austria) and centrifuged (800x*g*, 15min, no brake). The PBMC layer was collected carefully and washed (700x*g*, 5min, 4°C) with ice-cold isolation buffer (PBS supplemented with 2 mM ethylenediaminetetraacetic acid (Thermo Fisher Scientific, Waltham, USA) and 0.5% protease-free bovine serum albumin (Carl Roth, Karlsruhe, Germany). To remove remaining erythrocytes, a hypotonic lysis was performed by resuspending the cells in 20mL 0.2% sodium chloride (Merck KGaA, Darmstadt, Germany). 20 seconds later the same amount of 1.6% sodium chloride was added to restore isotonic conditions. After centrifugation (300x*g*, 10min, 4°C), cells were washed (425x*g*, 5min, 4°C) again with ice-cold isolation buffer.

### Low-Density Granulocyte Depletion

Low-density granulocyte depletion was performed using magnetic-activated cell sorting (MACS^®^) (Miltenyi Biotec, Bergisch Gladbach, Germany) as stated by the manufacturer. In detail, PBMC were pelleted, resuspended in isolation buffer (80µL per 10^7^ cells), cluster of differentiation (CD) 15 MicroBeads (Miltenyi Biotec, Bergisch Gladbach, Germany) were added (20µL per 10^7^ cells) and mixed well. Following a 15min incubation at 4°C, cells were washed by adding 30mL isolation buffer (425x*g*, 5min, 4°C) and resuspended in fresh isolation buffer (500µL per 10^8^ cells). Depletion was done using an AutoMACS™ Pro Separator (Miltenyi Biotec, Bergisch Gladbach, Germany) and the program “DepleteS”. The negative fraction (unlabeled cells) containing the PBMC was collected.

### Flow Cytometry

To verify the effectiveness of the depletion, 1x10^5^ cells before (patients with sepsis: n=3; healthy controls: n=11) and after depletion (patients with sepsis: d1 n=14; d8 n=12) were incubated with 5µL Human TruStain FcX™ (BioLegend, San Diego, USA) for Fc receptor blocking and stained by the addition of 5µL of each of the following antibodies: anti-CD3-fluoresceinisothiocyanate (FITC) (clone: UCHT1), anti-CD14-V450 (clone: MφP9), and anti-CD19-allophycocyanin (APC) (clone: HIB19) (all from BD Biosciences, Franklin Lakes, USA). After 30min incubation at 4°C in the dark, cells were washed (250x*g*, 5min) once with 2mL isolation buffer, resuspended in isolation buffer and directly measured. In addition, positive fractions from depletion (n=2) were stained and analyzed in the same manner.

For quantification of human leukocyte antigen-DR (HLA-DR) expression on CD14^+^ monocytes, 2x10^5^ cells were stained with 20µL anti-HLA-DR/anti-Monocyte PerCP-Cy5.5 reagent (clone: L243/MwP9) (BD Biosciences, Franklin Lakes, USA) for 30min in darkness and measured directly afterwards. BD Quantibrite PE tubes (BD Biosciences, Franklin Lakes, USA) were used for quantifying the average number of HLA-DR molecules per monocyte as indicated by the manufacturer.

For measurement of mitochondrial reactive oxygen species (ROS) production 1x10^5^ cells were washed with 2mL pre-warmed Hank’s Balanced Salt Solution (HBSS) and incubated with 5µM MitoSOX™ Red mitochondrial superoxide indicator diluted in HBSS (both from Thermo Fisher Scientific, Waltham, USA) for 10min at 37°C in darkness. Subsequently cells were washed with 2mL pre-warmed HBSS and resuspended in HBSS for measurement.

A FACSVerse™ flow cytometer (BD Biosciences, Franklin Lakes, USA) was used for all measurement. Results were analyzed using BD FACSuite™ software (BD Biosciences, Franklin Lakes, USA) and are shown using density plots.

### Cell Counting

Cells were counted after density gradient centrifugation and after depletion of low-density granulocytes by impedance-based particle detection using a Scepter™ 2.0 Cell Counter (Merck Millipore, Burlington, USA).

The total number of cells removed by the depletion process was calculated by subtracting the cell count after depletion (pure PBMC fraction) from the cell count before depletion (PBMC and low-density granulocytes).

### Cytokine Production

1.5x10^5^ cells each were resuspended in 300µL of Roswell Park Memorial Institute medium (Thermo Fisher Scientific Inc, Waltham, Mass), containing GlutaMAX, 100 units/mL penicillin, 100µg/mL streptomycin and 10% heat-inactivated fetal bovine serum ultra-low endotoxin (Cell Concepts, Umkirch, Germany). Stimulation was performed with 100ng/mL ultrapure lipopolysaccharide (LPS) (from *Escherichia coli*, strain O111:B4), 100ng/mL ultrapure flagellin (from *Pseudomonas aeruginosa*), 100µg/mL depleted zymosan (all from Invivogen, San Diego, Calif) or 3µL Dynabeads^®^ Human T-Activator CD3/CD28 (Thermo Fisher Scientific, Waltham, USA). Prior to use, Dynabeads were washed and resuspended in culture medium according to the manufacturer’s instruction. Control cells were incubated without a stimulating agent. Following a 24h incubation (37°C, 5% CO_2_), supernatants were collected by centrifugation (1,000x*g*, 5min) and interleukin-6 (IL–6) or interferon γ (IFNγ) levels were measured using colorimetric enzyme-linked immunosorbent assay (ELISA) (Human IL–6 and Human IFN-gamma Duo-Set ELISA; both from R&D Systems, Minneapolis, USA) according to the manufacturer’s instruction. Induced cytokine production was calculated as the difference between stimulation and control results.

### Statistical Analysis

Statistical analyses were performed in GraphPad Prism (V 8.0.2 for Windows, GraphPad Software, La Jolla, USA). Results are visualized as scatter plots. Horizontal lines depict the median. Statistical comparison between two groups was done using the Mann-Whitney test. Statistical significance was considered with *P* ≤ 0.05.

## Results

**Figure 1:**
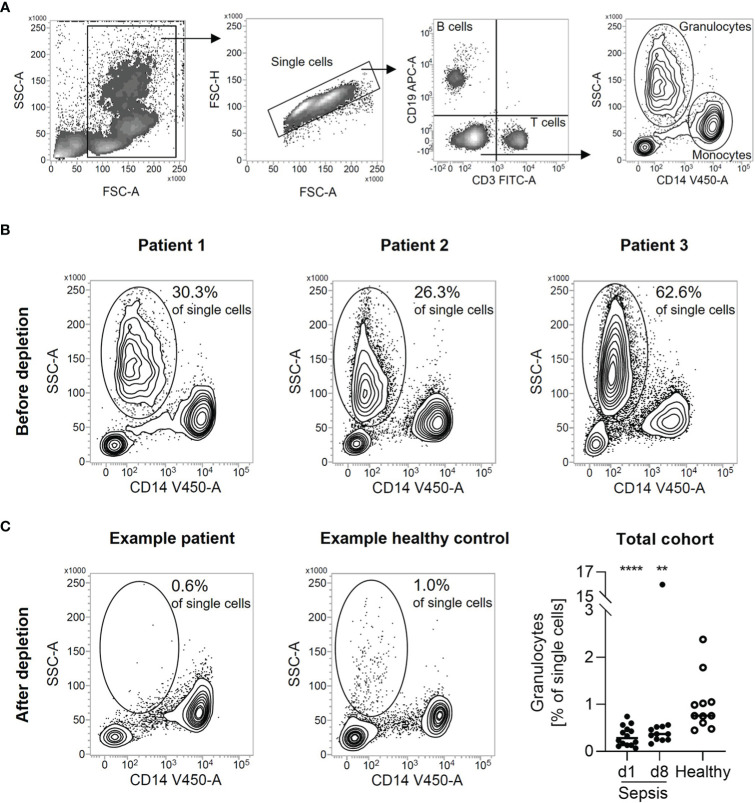
Depletion of CD15^+^ cells leads to pure PBMC from patients with sepsis. **(A)** Representative gating strategy for the identification of low-density granulocytes. Single cells were gated from all cellular events. B cells were identified as CD19^+^. T cells were identified as CD3^+^. Monocytes were identified as CD14^+^. Low-density granulocytes were identified as CD3^-^, CD14^-^, CD19^-^, SSC^hi^. **(B)** Granulocyte percentage in patients with sepsis after density gradient centrifugation. Density plots from three samples selected for analysis are shown. Numbers indicate the proportion of low-density granulocytes in all single cells. **(C)** Flow cytometric quality control of low-density granulocyte depletion. Representative density plots are shown for patients with sepsis after depletion of CD15^+^ cells from PBMC isolated by density gradient centrifugation respectively for healthy volunteers after density gradient centrifugation. Numbers indicate the proportion of low-density granulocytes in all single cells. For total cohort, relative percentages of low-density granulocytes after depletion of CD15^+^ cells are shown (in case of healthy volunteers after density gradient centrifugation). Each data point represents an individual patient (sepsis d1: n = 14; d8: n = 12/healthy: n = 11) and horizontal line marks the median. Group comparisons (sepsis *vs*. healthy) were performed using Mann-Whitney test (****P < 0.0001, **P ≤ 0.01).

Density gradient centrifugation is a widespread standard method to isolate PBMC in high purity from whole blood. We used this method to isolate PBMC from freshly drawn blood of patients with sepsis. Using flow cytometry to determine the composition of the isolated PBMC, we found a substantial amount of low-density granulocyte contamination. They were identified as granulocytes due to their size, granularity, and the absence of lineage markers for T cells (CD3), B cells (CD19), and monocytes (CD14) (**Figure 1A**). In three selected samples, 26.3%, 30.3%, and 62.6% of all single cells were found to be low-density granulocytes, respectively (**Figure 1B**). Thus, the PBMC’s purity is far from sufficient for subsequent experiments. Applying a bead-based depletion of CD15^+^ cells after the density gradient-based cell isolation resulted in the necessary purity of PBMC isolated from patients with sepsis. The remaining share of low-density granulocytes is even lower than in PBMC isolated from healthy controls by density gradient centrifugation only (**Figure 1C**).

**Figure 2:**
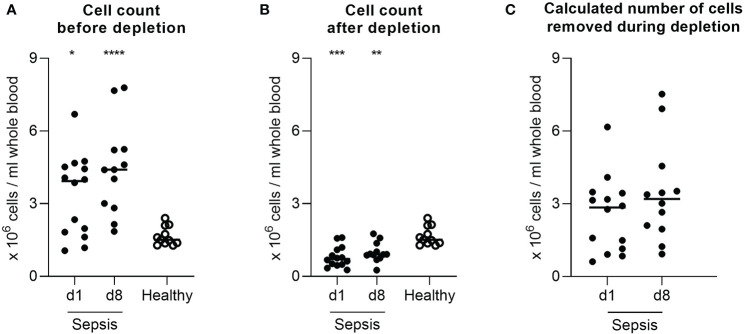
Low-density granulocytic contamination is also reflected in the number of isolated cells. Cell yield per mL blood after in case of patients with sepsis **(A)** density gradient centrifugation and **(B)** additional depletion of CD15^+^ cells, in case of healthy volunteers’ density gradient centrifugation. **(C)** Calculated total number of cells removed by the depletion process per mL blood in patients with sepsis. Each data point represents an individual patient (sepsis d1: n = 14; d8: n = 12/healthy: n = 11). Horizontal lines mark the median. Group comparisons (sepsis *vs*. healthy) were performed using Mann-Whitney test (****P < 0.0001, ***P ≤ 0.001, **P ≤ 0.01, *P ≤ 0.05).

**Figure 3:**
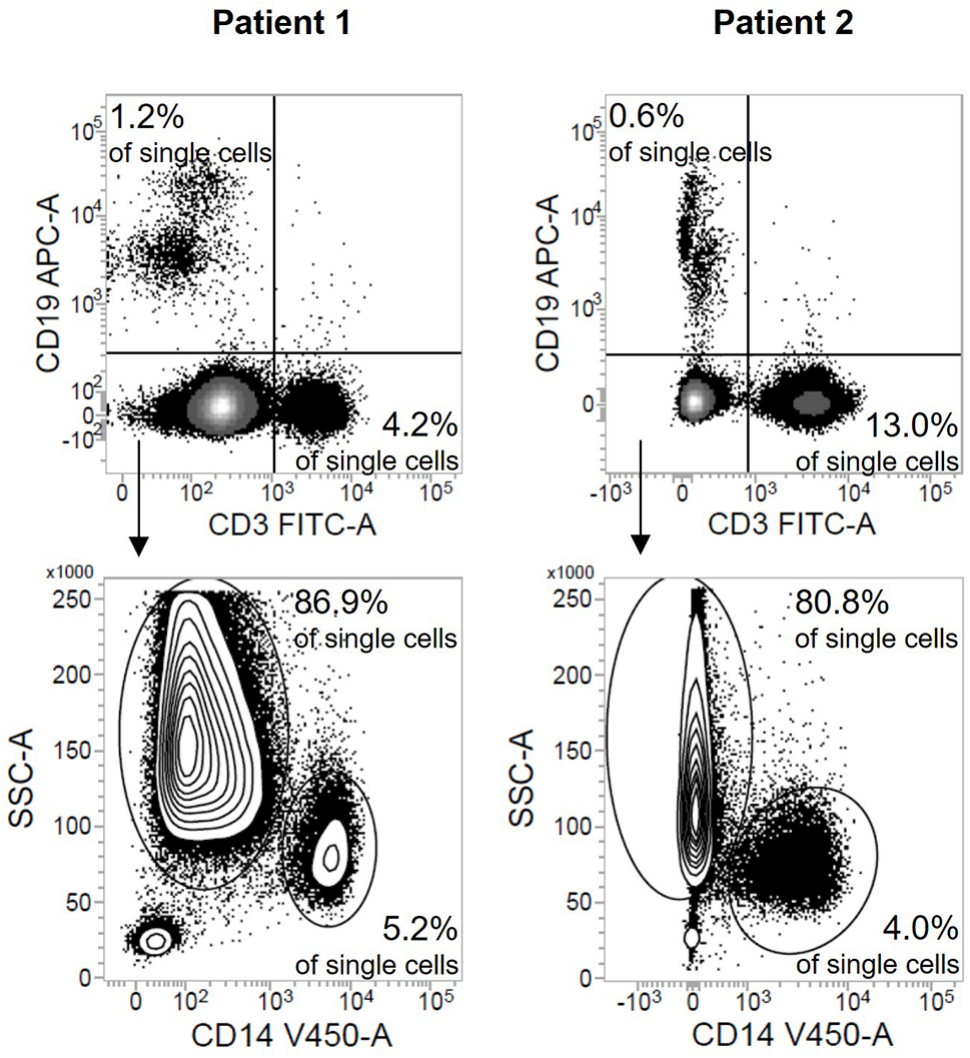
Depletion of CD15^+^ cells leads to a small loss of PBMC. Flow cytometric results after staining of the fraction containing the depleted CD15^+^ cells shown as density plots (sepsis: n = 2). Numbers indicate the proportion of B cells, T cells, monocytes, and low-density granulocytes respectively in all single cells.

The low-density granulocyte contamination is also reflected in the robust increased cell yield compared to healthy individuals after density gradient centrifugation (**Figure 2A**). The delta between this cell count and the value after depletion (**Figure 2B**) was used to computationally estimate the number of low-density granulocytes in the original sample emphasizing the high variability between different patients (d1: range 5.6x10^6^ cells; d8: range: 6.6x10^6^ cells) (**Figure 2C**). However, the actual low-density granulocyte amount is to be assumed lower, since PBMC are also lost during the depletion process. The magnetically labelled CD15^+^ cells were separated from all other, unmarked cells *via* retention in a column placed in a strong magnetic field. Apart from the low-density granulocytes sorted out as desired, we found also small proportions of B cells, T cells, and monocytes in this fraction (**Figure 3**).

**Figure 4:**
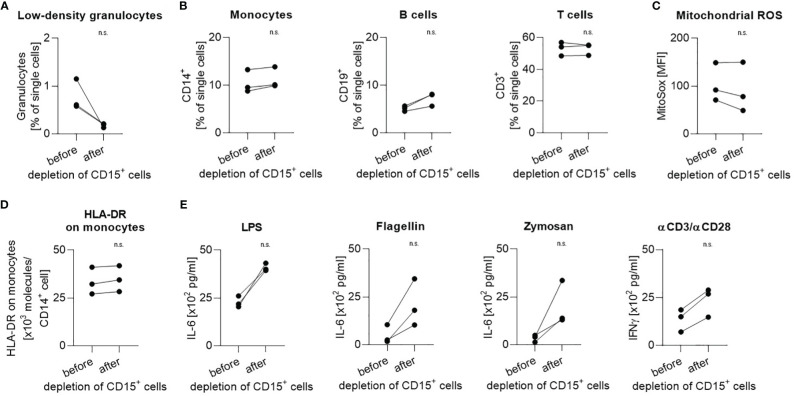
Handling during depletion has no impact on cellular function. Proportion of **(A)** low-density granulocytes, **(B)** monocytes, B cells, and T cells in all single cells before and after depletion of CD15^+^ cells. **(C)** Mitochondrial ROS expression level and **(D)** number of HLA-DR molecules per CD14^+^ monocyte before and after depletion of CD15^+^ cells. **(E)** Induced IL-6 production in response to stimulation with LPS, flagellin, or zymosan and induced IFNγ production in response to stimulation with human T-Activator CD3/CD28. Each data point represents a separate healthy volunteer (n=3). Paired results from the same donor before and after depletion of CD15^+^ cells are connected by a line. Group comparisons were performed using Mann-Whitney test (n.s., not significant).

To investigate a possible functional impact of the depletion process on the PBMC fraction, cells from three different healthy volunteers were isolated by density gradient centrifugation but only from half of the PBMC fraction CD15^+^ cells were depleted. Since healthy people also possess an, albeit very low, proportion of low-density granulocytes in their blood (**Figure 4A**, range 0.5% - 1.2% of single cells; **Figure 1C**, range 0.4 – 1.6% of single cells), the depletion of these cells led to slight shifts in the ratio of individual fractions to the total amount of PBMC (**Figure 4B**). The expression of mitochondrial ROS stayed constant in comparison before and after depletion (**Figure 4C**). Moreover, the number of HLA-DR molecules on the surface of CD14^+^ monocytes remained stable (**Figure 4D**). When identical cell numbers were stimulated with LPS, flagellin, zymosan, or human T-Activator CD3/CD28, the depleted fractions secreted higher cytokine amounts than the non-depleted fractions (**Figure 4E**).

## Discussion

We here report the efficacy of magnetic bead based CD15^+^ cell depletion to gain highly pure PBMC from freshly drawn blood samples of septic patients. While we used an automated cell separation device for our study, manual single-use separators might be a feasible alternative option to implement this method without extensive costs.

Preobrazhensky and Bahler reported a comparable immunomagnetic approach removing granulocyte contamination from cryopreserved blood from healthy individuals (17). Additionally, CD15 is a well-established marker for neutrophils, the largest granulocyte population by distance in the blood of patients with sepsis, and described as being expressed on low-density granulocytes in a wide variety of diseases (11, 12, 14, 18, 19). Therefore, it seems persuasive that depletion of CD15^+^ cells is a successful strategy regardless of the source and function of contaminating low-density granulocytes or the underlying pathological condition.

The observed loss of PBMC might be technical. As CD15 is expressed not only on neutrophils and eosinophils but to varying degrees also on monocytes, a fraction of these cells is depleted with our approach, too. On the other hand, a disease-related interaction between column-bound granulocytes and cells contained in the PBMC fraction might prevent the latter from passing the sorting column. Moreover, the selected separation program (“DepleteS”) is optimized by the manufacturer to achieve a highly pure fraction of unlabeled cells. To this end, it is accepted that a small proportion of unlabeled cells end up in the labelled fraction and thus being lost. The PBMC loss is, however, negligible compared to the achieved high purity of PBMC, especially since the proportion of low-density granulocytes varies greatly between individual patients and is therefore a highly variable bias that is difficult to control. Nevertheless, the well-known phenomenon of sepsis-associated lymphopenia (20) is the root cause for the significantly lower PBMC yield per milliliter blood from patients with sepsis compared to healthy volunteers.

The co-purifying low-density granulocytes are phenotypically different from mature neutrophils displaying characteristics of immature neutrophils (8) and possess myeloid derived suppressor functions (19). Interestingly, Darcy et al. reported that the number of these cells depends on the severity of the disease. This is an explanation for our observation, that the number of low-density granulocytes co-purifying with the PBMC fraction varies highly between patients. Although some of the studies depicting the problem of contaminating, co-purifying low-density granulocytes have been published a decade ago, it is still common practice today to use density gradient centrifugation for PBMC isolation from patients with sepsis (21–33). This goes along with a lack of reported information on the isolation purity and identification of the contained cell types. Partly contradictory results between different studies might be explained by clinical differences in the study populations (e.g., severity), not only leading to divergent compositions of PBMC, but primarily to a different proportion of low-density granulocyte contamination. Furthermore, this high share in samples of patients strongly limits the biological comparability with PBMC isolated from control groups (e.g., healthy individuals) without such high abundance of co-purifying low-density granulocytes and, thus, a widely differing cell distribution.

By comparing cells from healthy volunteers, which have a very low proportion of low-density granulocytes in their blood, before and after depletion of CD15^+^ cells, we can show that the external magnetic field and the additional shear stress caused by the necessary pipetting during the depletion process have a negligible technical impact on the PBMC and do not compromise functional cellular properties. The stable expression levels of mitochondrial ROS between pre- and post-depletion indicate that the cells are not further activated during the depletion process. Antigen presentation, a key function of monocytes and essential for proper activation of the adaptive immune system, is not impaired, as shown by stable HLA-DR expression on CD14^+^ monocytes. As expected, the shares of individual PBMC fractions are slightly altered because the low-density granulocytes, although present in small numbers, are removed. This granulocyte subpopulation has myeloid derived suppressor functions, as discussed above. The depletion-related loss of this function together with the higher proportion of cytokine-producing cells after depletion, while the absolute number of stimulated cells remained the same, account for the higher cytokine amounts after depletion. These comparatively higher amounts thus arise less from altered function of the cytokine-producing cells themselves, but rather are a side effect of the deliberate loss of CD15^+^ cells. This uniform effect weighs small compared to the heterogenous influence of the strongly varying proportions of co-purifying granulocytes, as this high variability between individual patients impair the reproducibility and comparability between different studies and towards controls groups. However, the approach we propose here may not be suitable for all experimental settings. A limitation of our study is the lack of direct evidence that depletion of CD15^+^ cells does not affect the function of individual PBMC fractions even in patients with sepsis. This needs to be investigated in further studies and adapted to the particular study designs. Moreover, our experimental design does not allow us to assess whether the slight loss of PBMC during depletion is stochastically random or whether cells with specific functions that affect the interaction with low-density granulocytes are primarily lost. Notwithstanding, our results emphasize the necessity of a high degree of protocol standardization across all samples and importantly also study groups within a clinical study to keep sample-handling-induced bias uniform and as low as possible.

Combining density gradient centrifugation and CD15^+^ cell depletion is a simple and effective strategy to gain highly pure, functionally intact PBMC from patients with sepsis. Eliminating this inter-patient highly variable bias contributes to a higher quality and reliability of results as well as a better comparability between different studies.

## Data Availability Statement

The raw data supporting the conclusions of this article will be made available by the authors, without undue reservation.

## Ethics Statement

The studies involving human participants were reviewed and approved by the ethics committee of the Medical Faculty of the Heidelberg University. The patients/participants provided their written informed consent to participate in this study.

## Author Contributions

All authors contributed to the study design. MO, SU, MW, and FU obtained ethics approval. MO, SU, and MW performed patient recruitment and informed consent. JS and FU established laboratory methodology, performed data collection and analysis, and wrote the manuscript. All authors contributed to the article and approved the submitted version.

## Funding

The SEPSDIA project (reference number 11501) has been conducted within the framework of the European funding program “Eurostars”. The German consortium partner has received financial support from the German Federal Ministry of Education and Research (BMBF), Berlin.

## Conflict of Interest

The authors declare that the research was conducted in the absence of any commercial or financial relationships that could be construed as a potential conflict of interest.

## Publisher’s Note

All claims expressed in this article are solely those of the authors and do not necessarily represent those of their affiliated organizations, or those of the publisher, the editors and the reviewers. Any product that may be evaluated in this article, or claim that may be made by its manufacturer, is not guaranteed or endorsed by the publisher.

## Abbreviations

APC, allophycocyanin; CD, cluster of differentiation; ELISA, enzyme-linked immunosorbent assay; FACS, fluorescence-activated cell sorting; FITC, fluorescein isothiocyanate; HBSS, Hank's Balanced Salt Solution; HLA-DR, human leukocyte antigen-DR; IL, interleukin; IFN, interferon; LPS, lipopolysaccharide; MACS, magnetic-activated cell sorting; PBMC, peripheral blood mononuclear cells; PBS, phosphate-buffered saline; ROS, reactive oxygen species; SIRS, systemic inflammatory response syndrome; SOFA score, sequential organ failure assessment score.
